# Decoration Increases the Conspicuousness of Raptor Nests

**DOI:** 10.1371/journal.pone.0157440

**Published:** 2016-07-25

**Authors:** David Canal, Margarita Mulero-Pázmány, Juan José Negro, Fabrizio Sergio

**Affiliations:** 1 Department of Evolutionary Ecology, Doñana Biological Station – CSIC, Av. Américo Vespucio s/n, 41092 Sevilla, Spain; 2 Universidad Técnica Particular de Loja, Departamento de Ciencias Naturales, San Cayetano Alto, Loja, Ecuador; 3 Department of Conservation Biology, Doñana Biological Station – CSIC, Av. Américo Vespucio s/n, 41092 Sevilla, Spain; CSIC-EEZA, SPAIN

## Abstract

Avian nests are frequently concealed or camouflaged, but a number of species builds noticeable nests or use conspicuous materials for nest decoration. In most cases, nest decoration has a role in mate choice or provides thermoregulatory or antiparasitic benefits. In territorial species however, decorations may serve additional or complementary functions, such as extended phenotypic signaling of nest-site occupancy and social status to potential intruders. The latter may benefit both signaler and receiver by minimizing the risk of aggressive interactions, especially in organisms with dangerous weaponry. Support for this hypothesis was recently found in a population of black kites (*Milvus migrans*), a territorial raptor that decorates its nest with white artificial materials. However, the crucial assumption that nest decorations increased nest-site visibility to conspecifics was not assessed, a key aspect given that black kite nests may be well concealed within the canopy. Here, we used an unmanned aircraft system to take pictures of black kite nests, with and without an experimentally placed decoration, from different altitudes and distances simulating the perspective of a flying and approaching, prospecting intruder. The pictures were shown to human volunteers through a standardized routine to determine whether detection rates varied according the nest decoration status and distance. Decorated nests consistently showed a higher detection frequency and a lower detection-latency, compared to undecorated versions of the same nests. Our results confirm that nest decoration in this species may act as a signaling medium that enhances nest visibility for aerial receivers, even at large distances. This finding complements previous work on this communication system, which showed that nest decoration was a threat informing trespassing conspecifics on the social dominance, territory quality and fighting capabilities of the signaler.

## Introduction

Among vertebrates, birds are renowned for their construction and architectural capabilities. They build nests as containers for their offspring and/or as roosting sites, and may invest a substantial amount of time and energy in building activities, even if many are ephemeral structures that last only a few weeks [1]. Avian nests are often concealed or camouflaged because individuals are frequently vulnerable to attacks by predators or aggressive conspecifics when incubating eggs or brooding nestlings [2,3]. Accordingly, behaviors and nest designs that minimize predation risk and/or brood failure are favored by natural selection. Thus, birds choose carefully the location and design of their nests in relation to the presence and density of predators [4,5] and any element that may increase conspicuousness, such as the whitish faecal sacs excreted by the nestlings of passerine species, tend to be removed readily by the attending parents [6].

Although crypsis seems to be sought by a large proportion of species, a number of taxa use conspicuous (e.g. artificial) materials for nest building or decoration, but the adaptive function of this behavior is still poorly understood (review in [7]). Typically, nest decorations have been suggested to act as extended phenotype signals in courtship displays. For instance, the addition of ornamental feathers outside the nest cup by female spotless starlings (*Sturnus unicolor*) or male blue tits (*Cyanistes caeruleus*) seems to have a role in sexual selection [8]. Conversely, in other species, marking the nest might be a way to advertise nest-site occupancy and social status (i.e. territoriality) to potential intruders, even in the absence of the owners [7]. Under this scenario, only high quality owners would expose their nest and assume the costs of aggressive encounters with conspecifics that may covet the territory, thus ensuring the reliability of the signal [9]. These costs were shown to hold true for a population of black kites (*Milvus migrans*), a raptorial bird that decorates the nest with white artificial materials [10]. In such study, the authors showed through observational data and multiple manipulative experiments that nest decoration: (1) was minimum for youngest individuals, peaked for individuals in prime age (7–11 years old) characterized by maximum reproduction and survival, and declined with senescence thereafter; (2) it acted as an honest signal informing conspecifics on the viability, territory quality, fighting ability and social dominance of the signaler; and (3) low quality individuals refrained from dishonest signaling due to the high costs associated with it (increased aggressive intrusions and risk of depredation), thus ensuring the reliability of the signal. However, given that black kite nests are often well hidden in the canopy, the crucial hypothesis that nest decorations increased nest-site visibility to other kites, which was assumed to be true, must be specifically tested to confirm that nest decoration could function as a reliable signal of threat against intruders.

Black kites build platform nests composed of tree sticks and branches. These open structures are lined up with finer materials such as twigs, soil residues or mud, and decorated just before egg laying by adding artificial materials, such as plastics or paper. Nest sites are often reused for several years in succession and new materials added every breeding season. In a previous study, decoration was rated as the percentage of the nest surface covered by non-natural fabrics, such as plastic or paper [10]. The rating was done by an observer who could not consider the actual visibility or conspicuousness of the nest-site as perceived by a flying intruder and that can be affected by factors such as nest size or tree architecture, among others. Thus, to fully test the hypothesis that decorated nests may function as signaling devices to trespassing conspecifics, it is essential to demonstrate that ornamented nests are indeed more visible from the air than they would be without decoration. The implications of such a demonstration are vast, because evidence for social status signaling through nest marking is very scarce in birds (e.g. [11]).

In this study, we used state-of-the-art UAS (Unmanned Aircraft Systems) technology to simulate the aerial perspective of trespassing, flying black kites, and assess whether decorated nests were more conspicuous than undecorated ones to a human observer. To this end, we flew at pre-determined distances from actual nests built by black kites a UAS, equipped with a digital high-resolution camera, and gathered images of the nests with and without an experimentally placed decoration. The images were later standardized using ad hoc prepared software and shown to volunteers through a standardized routine to determine whether detection rate varied according to nest decoration status and distance.

## Materials and Methods

### Ethics statement

The study was conducted in accordance with EC Directive 86/609/EEC for animal experiments, and with the current Spanish legislation involving aviation safety. Non-invasive methods were used during experiments. No animals were sacrificed, handled nor sampled and thus, animal ethic evaluation was not required. Field technicians had the required licenses to operate in the frequencies used for this work. All the field procedures were reviewed and accepted by the authority yielding the permit (Doñana National Park authorities, Junta de Andalucía; permit reference: 3236/FQH/mdcg).

### Nest inspections

Field work was conducted in Doñana National Park (Spain: 37° 6’ N, 6° 28’ E) at the beginning of May 2014, when tree foliage is consistent with the average situation experienced by kites during the peak of territorial intrusions (trespassing) by non-breeding individuals attempting territory takeovers. By this date, most individuals were in the pre-laying or incubation stage of the breeding cycle (the mean laying date of the population is 15 April, but may range from March to mid-June), the periods with the highest decoration intensity and trespassing rates in the population [10]. A total of 15 nests were randomly selected for inspection within the Park, covering the most representative areas, tree types and range of nest-exposures (from very exposed to hidden in the canopy). Nests with eggs or chicks were excluded from the study to avoid disturbance, except for one nest that contained cold, already abandoned eggs, which were removed for contaminant-analysis before the flights and thus did not interfere with the experiment.

During each UAS flight-session, the target nest was climbed, cleaned of any decoration material if already present and then assessed through UAS-flights twice, i.e. with and without being decorated by us with a white plastic bag. A white plastic bag was chosen because this is by far the most preferred (in relation to other colors and materials) and commonly used item (90% occurrence) by black kites in Doñana to decorate their nests [11]. The tree-climber, experienced with checking hundreds of kite nests in previous years, was instructed to place the white plastic bag so as to simulate a high level of nest decoration, as observed in real kite nests (e.g. see photo in Fig 3b of [11]). Thus, when decorated, the white bag covered 60–80 percent of the nest platform corresponding to a high-level of nest decoration [10]. This value was chosen based on the following reasons: (1) Financial and logistical constraints limited the sample size to a maximum of 15 nests and precluded the possibility to examine how visibility varied along a gradient of decoration intensity. Therefore, we decided to focus on a restricted range of decoration-levels corresponding to individuals that make the active decision to decorate their nest and benefit from it (see below). (2) In this sense, the basic question of the study was: “for a kite that decorates the nest, does this increase its potential visibility to other kites?”. Based on previous experiments, the portion of the population that most clearly, decorate the nest is composed by individuals in prime age [10]. These are the ones that most frequently collected experimental decorations when offered by the researchers [10], and, among those that collected these experimental items, more than half had decoration levels of 60–80%. (3) Individuals that decorate the nest profusely (i.e. at the end-tail of the decoration range) are those that will most benefit by the signal in terms of lowered intrusion rates and fights against challengers, as shown in [10]. Thus, they would be the ones most interested in “designing” a decoration configuration that is visible from as far as possible (otherwise, by the time a conspecific sees the decoration, it may be too late to avoid the aggressive encounters due to the intrusion). (4) Finally, by focusing on high-decoration levels, the analysis of the basic question outlined above was conservative: if nest decoration, as simulated here, does not increase nest visibility and detection, then it would be even less likely to do so for lower levels of decoration.

In addition to the manipulation, each nest was characterized by its dimensions (length and width), measured in cm with a ruler, and “opening angles”, defined as the three angles of unobstructed view of the sky, measured with a compass from the center of the nest-cup outwards: (1) to the right in horizontal, compared to the North; (2) to the left in horizontal, compared to the North; and (3) vertically, compared to the level of the platform. This characterized the potential breadth of visibility of the nest from the outside of the tree both vertically and horizontally.

**Fig 1:**
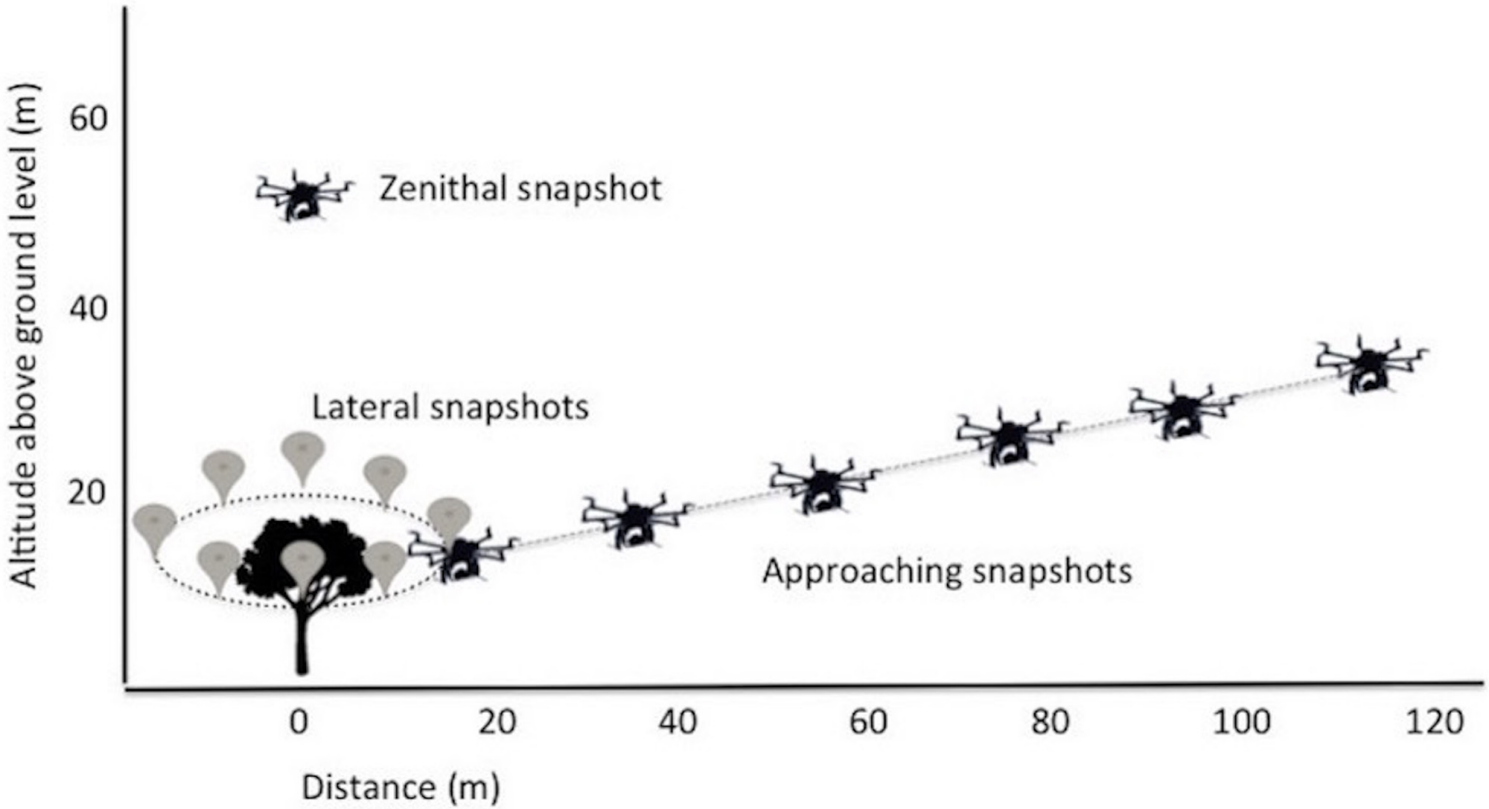
Illustration of the flight plan used during the experiments. The hexacopter first ascended to 50 m above ground level and took one zenithal image (zenithal snapshot), then flew at 15 m radius 360° around the tree and at 10 m AGL, taking regularly photos (lateral snapshots). Finally, the hexacopter approached the nest making a series of photos (approaching snapshots) at distances of 115, 95, 75, 55, 35, 15 m (30, 26, 22, 18, 14, 10 m AGL, respectively), simulating the aerial perspective of an approaching black kite that intends to inspect the nest.

We performed two flights per nest (one with and one without artificial decoration) using a hexacopter to photograph them (with a Sony Nex5 camera, 16 Megapixels resolution) at different distances and angles (Fig 1 and Figure A in S1 File). All nests were assessed following the same sequential procedure: (1) first, we took one zenithal image (“zenithal snapshot” hereafter), hovering at 50 m above ground level (AGL hereafter). (2) Then, the hexacopter flew at 10 m AGL around the tree, designing a circle of 15 m radius 360° and making a series of eight regularly-spaced photos of the treetop (hereafter “lateral snapshots”). Finally, the hexacopter flew away from the nest and then approached it making a series of photos (hereafter “approaching snapshots”) at distances of 115, 95, 75, 55, 35 and 15 m (at 30, 26, 22, 18, 14 and 10 m AGL, respectively; Fig 1). This sequence of declining distances and heights was chosen to simulate the progressive approach of a trespassing, prospecting black kite that intends to inspect the content of a nest. In Doñana, kites breed at high density in loose aggregations with small inter-nest distances (e.g. [12]), so that, on average, a prospecting trespasser would start to enter the defended area and elicit the aggressive response by the owner between 30–120 m from a nest. Thus, the sequence of distances of the approaching snapshots covered the gradient ranging from approaching the border of a territory from its outside to a full intrusion and mimicked the actual behavior observed during trespassing, although simplified into a straight-line flight.

We randomly alternated the sequence of decoration treatments within nests to minimize the potential effect of light conditions on the detectability of the nests. All flights were performed in daylight between 7:30 and 21:00h (local time) by a professional team of operators and field coordinators from Flying-Cam (http://www.flying-cam.com). Each nest-assessment required around 1.5 hours of overall work (approximately 15–20 min to climb the tree and measure the nest characteristics, 45–50 min of active flight and 20–30 min of material download, assemblage, testing and reload on vehicles) to complete all recordings (of the decorated and non-decorated treatments), while moving from one nest to the next required ~ 20–60 min. Flights were performed on autonomous mode guided by the on-board autopilot that followed a GPS waypoints-trajectory (pre-programmed in the previous days based on the GPS position of the nest location), but keeping the capability for the human pilot to take control of the aircraft in the eventuality of danger. The take-off and landing were performed in manual mode. A second operator using real time video from the UAS adjusted the gimbal and remotely controlled the onboard camera to perform the snapshots of the nests. All the images were associated to their GPS coordinates and barometric altitude extracted from the flight tracks.

### Preparation of nest images for visibility trials

**Fig 2:**
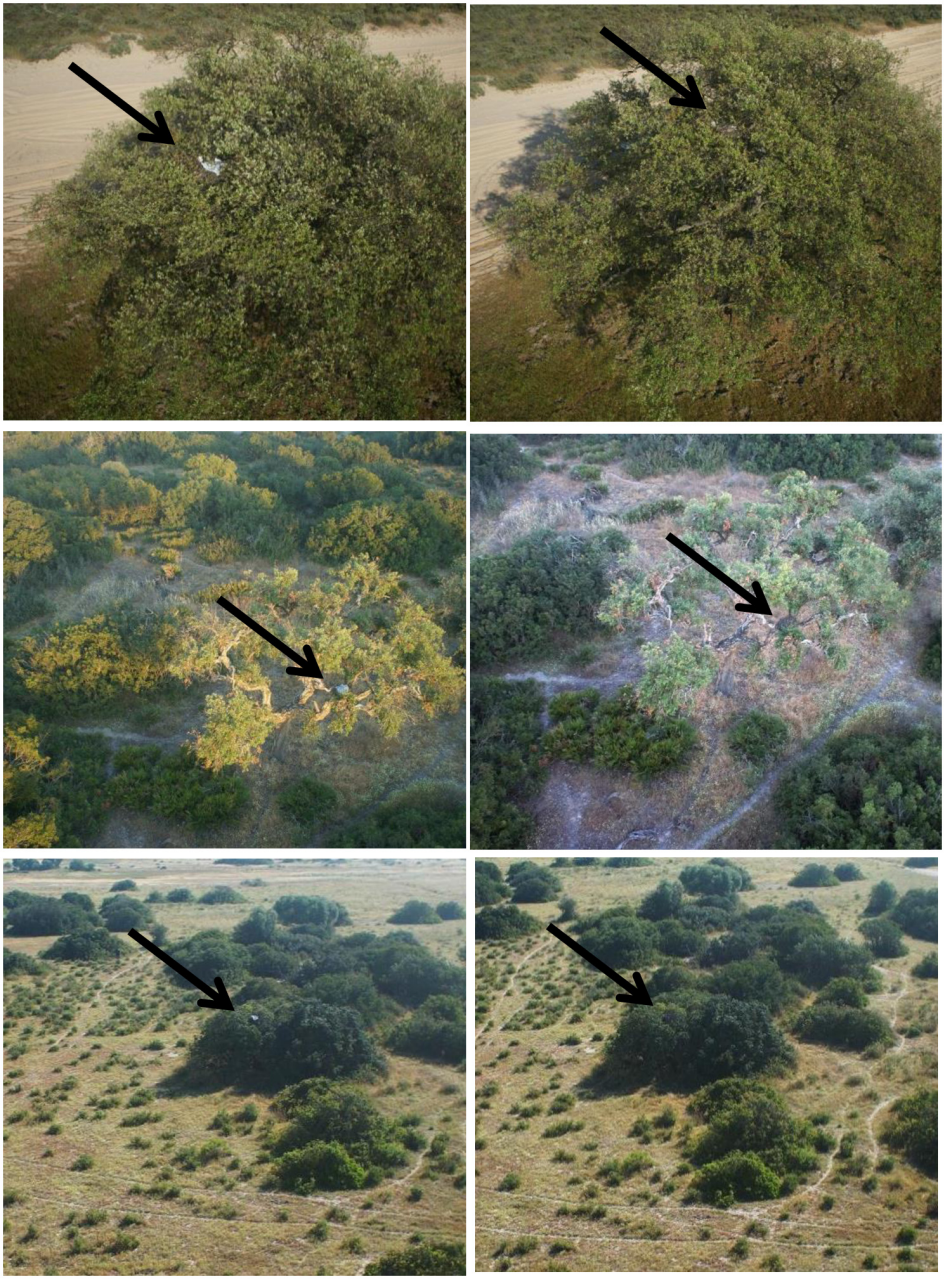
Images taken by the hexacopter of black kite nests experimentally decorated by the researchers (left side) and of the same nest without decoration (“self-control nest”, right side). The experimental decoration covered ca 80% of the nest platform, corresponding to a high-level of decoration in real nests, typical of birds in prime age (see [10]). Black arrows indicate the position of the nests. The images have been cropped and enlarged for illustrative purposes.

We took 662 images from the 15 nests inspected with and without decoration (Figs 1 and 2). Preliminary trials by DC and MM showed that finding nests in the pictures taken at distances of 95 and 115m (26 and 30m AGL, respectively) during the approaching flights had a very low detection rate and thus, those images were removed for further analyses. Before performing the definitive trials, all images were revised and, if necessary, treated with Photoshop CS6 (Adobe, San Jose, CA, USA) to remove features potentially facilitating the detection of nest location (for example, the car in the nest proximity).

To estimate the detectability of black kite nests to trespassers, we conducted trials of nest detectability using 25 volunteers as "experimental conspecifics”. The assumption behind such approach is that, if decoration makes a nest more visible to a human observer, it will make it even more visible to the keener vision of a raptorial bird [13]. To make the assessment by volunteers more rapid and standardized, we developed a Java program that projected the images on a laptop computer in a predetermined order (see below) and asked each observer to try to detect one nest per image and click on its presumed location. The program allowed 15 seconds to the observer for locating the nest, recorded the latency to locate it and assessed if the “clicked area” matched the real nest location. Each time an observer clicked on an image, the program switched to the next one, regardless of whether the volunteer succeeded or not in correctly detecting the nest and without feedback about their success in locating the nests. Also, if the observer did not click on the image within 15 seconds, the nest was recorded as non-detected and the program switched to the next photograph. Previous to the trials, we conducted a training session with 10 pictures that portrayed real kite nests and provided feedback to the participants, so as to accustom them to the general appearance of the target object of their search and standardize as much as possible the search image across participants. The images shown during the training session (15 seconds per image) were the same for all the participants and none of them were used in the subsequent trials. The observers had no previous knowledge of the aims of the study and none participated more than once in the experiment.

Each observer visualized 150 images of the 15 experimental nests divided in two sets. In the first set, consisting of 60 images, the observer was exposed to: (i) a pair of zenithal images of each nest (with and without decoration) taken at 50 m AGL, and (ii) a pair of lateral images of each nest (with and without decoration) taken circularly around the nest at 15 m radius and 10 m AGL. In the second set of images, formed by a total of 90 photographs, the volunteers visualized three pairs of approaching snapshots per nest (for the 15 decorated and 15 non-decorated nests), ordered from farther to closer (75 m, 55 m and 35 m distance), thus simulating the progressive approach of a trespassing black kite that intends to inspect the content of the nest.

Cumulatively, each volunteer dedicated approximately 25 minutes to visualize all the images. The images projected and their relative order varied between trials as, for example, there was more than one lateral image available for the 10 m AGL snapshot. To minimize memory-biases (e.g. nest location and characteristics of the surrounding landscape), the pictures from the same nest (regardless of its decoration treatment) were separated from each other by at least four photographs. Similarly, in the case of the approaching flights, the series of approaching snapshots for the same nest (i.e. from the decorated and non-decorated treatment) were separated from each other by at least four series of photographs. Finally, we randomized the position of the nest within the images by cropping each photograph to the size of 4175 x 2774 pixels (a 15% of reduction from the initial resolution of 4912 x 3264 pixels) in Photoshop CS6. In this way, we reduced the habitat similarity between images of the same nest. Based on pre-trials, we are confident that such procedures prevented any substantial effect of memory-biases, which would be homogenous across treatments and observers anyway. All trials were conducted using the same screen (HP ZR22w, 22 inches, 1920 x 1080 resolution) positioned at a distance of 50 cm from the observer.

### Statistical analyses

We used generalized linear mixed models (GLMM) to test whether (i) the probability of nest detection (0/1; binomial distribution, logit link function) and (ii) the latency to nest detection (time elapsed between initial image projection and nest detection, more details below; Gaussian distribution) was related to the decoration treatment (decorated *vs* non-decorated), the distance of the hexacopter to the nest and/or the interaction between nest decoration and distance. “Nest identity” and the “identity of the volunteers” were included in all the models as random factors. In the models on latency, we only included images in which the nests were detected by the participant in both treatments. Previously to running these models, latency to nest detection (max = 15 seconds) was log-transformed to satisfy the assumptions of normality. We built separate models to analyse zenithal, lateral and approaching snapshots. Additionally, in the case of the approaching snapshots, we ran models including only the first ‘correct detection’ of each nest (i.e. 2 images per nest, one per treatment) instead of the 3 consecutive images per treatment. For example, if Volunteer A detected nest X in the 75 m distance photo, then all subsequent detections of nest X by A (regardless of the decoration treatment) were not included in the analyses. Results remained unchanged (see Table A in S1 File) for both the probability of nest detection and latency to nest detection.

Selection of the minimum adequate models was done by sequentially dropping non-significant terms from fully saturated models (containing all main effects and interactions) in a hierarchical way, starting with the least significant terms. We performed likelihood ratio tests to confirm whether the inclusion of a predictor was significantly informative. In these tests, the full model, including the focal predictor, is compared with its restricted counterpart without the same predictor, and the significance of the predictor is obtained by a chi-square distribution.

We systematically performed model diagnostics statistics while modelling to avoid misleading conclusions based on statistical artifacts. To this end, we checked assumptions about the distribution of residuals through diagnostics plots and examined collinearity. These analyses did not show any obvious deviation from GLMM assumptions or any collinearity problems. Statistical analyses were implemented in R 3.1.2 (R Development Core Team 2015) with the package lmerTest [14].

## Results

**Fig 3:**
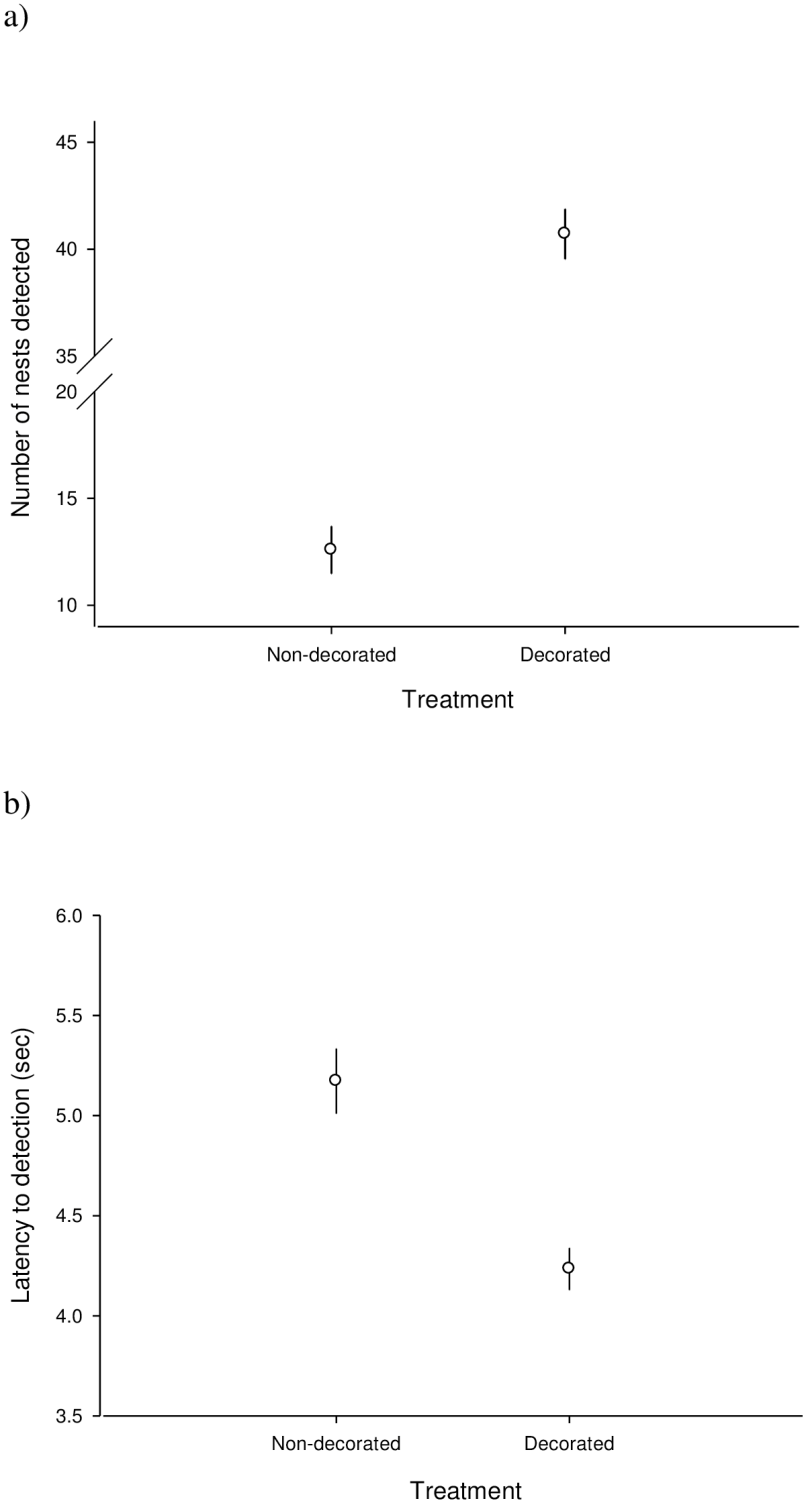
a) Mean number (± SE) of nests correctly detected by volunteers in standardized surveys of images captured by the hexacopters, and b) mean latency (± SE) to their detection in relation to the decoration treatment (decorated vs non-decorated nests; n = 150 images assessed by 25 volunteers for 15 nests, each measured as decorated or not). Bars represent 1 SE.

Overall, the group of decorated nests had a higher detection frequency and a lower latency to detection than the non-decorated nests (GLMM: number of detected nests: p < 0.001; Latency to detection: p = 0.027, Fig 3). The effect of nest dimensions and nest “opening angles” on the probability and time to detection was never significant (GLMM: all p > 0.18).

**Fig 4:**
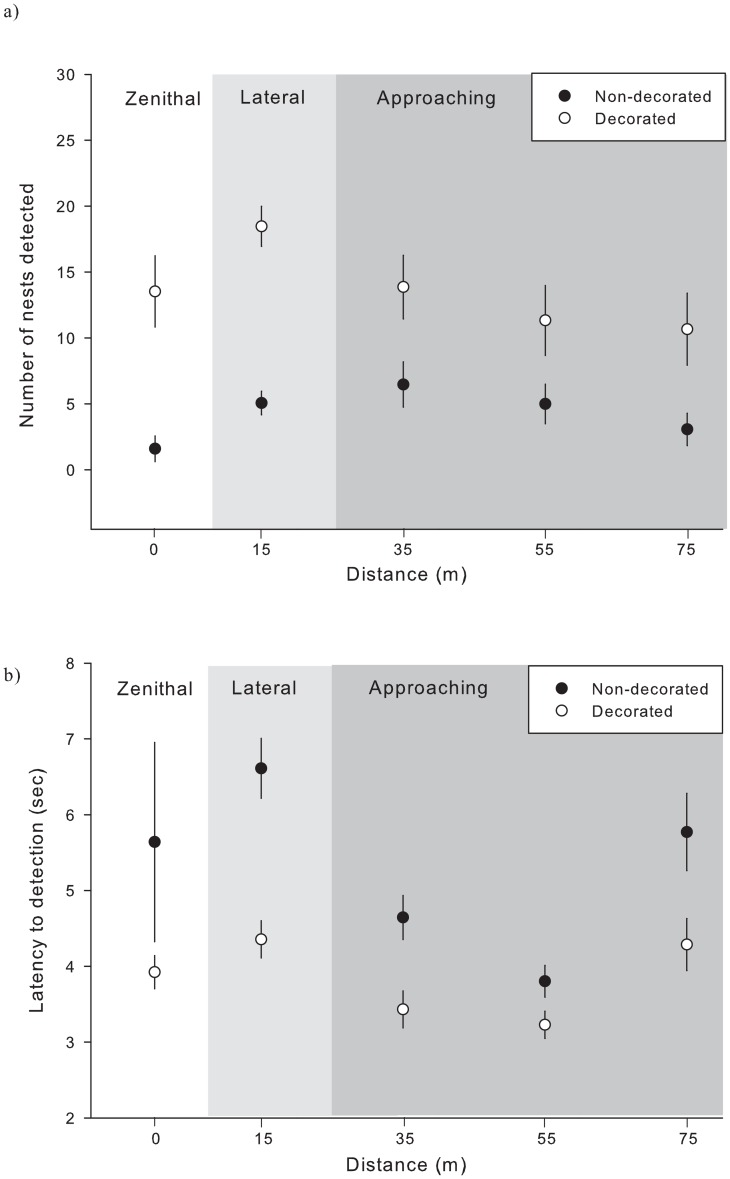
a) Mean number (± SE) of nests correctly detected by volunteers and b) mean latency (± SE) to their detection in relation to the position of the hexacopter (zenithal, lateral and approaching snapshots) when capturing the image and the decoration treatment (decorated vs non-decorated nests; n = 30, 30 and 90 images from zenithal, lateral and approaching snapshots, respectively, assessed by 25 volunteers for 15 nests, each measured as decorated or not).

Once we experimentally decorated a nest, its detection probability increased significantly and its latency to detection declined (Tables 1, 2 and Fig 4). This held true for all set of images, i.e. zenithal, lateral and approaching snapshots (Fig 4). In addition, for the set of approaching snapshots, the latency to nest detection depended on the interaction between treatment and distance. At shorter (35m) and medium (55m) distances, the latency to detection, although always lower in decorated nests, varied similarly in both decorated and non-decorated nests. With further increasing distances (75m) however, the latency to detection showed a steeper increase in non-decorated nests (Fig 4).

**Table 1:** Nest detection probability by human observers in relation to decoration treatment (decorated and non-decorated) and distance to the nest. Three models were run to analyses independently the detectability of the nests from: zenithal snapshots (50m AGL), lateral snapshots (15m and 10m AGL) and approaching snapshots (75, 55, 35m and 22, 18, 14m AGL) respectively.

	Estimate	Std. Error	z value	P
**Zenithal snapshot**				
Intercept	-5.539	0.938	-5.9	<0.001
Treatment (decorated)	6.04	0.608	9.924	<0.001
**Lateral snapshot**				
Intercept	-1.99	0.329	-6.053	<0.001
Treatment (decorated)	3.575	0.267	13.362	<0.001
**Approaching snapshot**				
Intercept	-1.035	0.723	-1.431	0.152
Treatment (decorated)	2.612	0.158	16.507	<0.001
Distance	-0.033	0.006	-5.882	<0.001
Treatment*Distance	0.011	0.008	1.299	0.194

**Table 2:** Latency to nest detection by human observers in relation to decoration treatment (decorated and non-decorated) and distance to the nest. Three models were run to analyses independently the detectability of the nests from: zenithal snapshots (50m AGL), lateral snapshots (15m and 10m AGL) and approaching snapshots (75, 55, 35m and 22, 18, 14m AGL) respectively.

	Estimate	Std. Error	t value	P
**Zenithal snapshot**				
Intercept	1.818	0.067	27.194	<0.001
Treatment (decorated)	-0.522	0.085	-6.159	<0.001
**Lateral snapshot**				
Intercept	1.816	0.105	17.242	0.001
Treatment (decorated)	-0.364	0.057	-6.405	<0.001
**Approaching snapshot**				
Intercept	1.179	0.188	6.252	<0.001
Treatment (decorated)	-0.662	0.159	-4.156	<0.001
Distance	0.018	0.011	1.747	0.093
Treatment*Distance	0.022	0.009	2.485	0.0135

## Discussion

This is the first study that attempts to directly estimate nest detectability from a bird’s perspective based on human vision using UAS technology. Using such tools, we experimentally show that the presence of ornamental white plastic in black kite nests increase their conspicuousness, even at large distances, compared to undecorated versions of the same nests. These findings, in combination with previous work on this system [10], support the hypothesis that the nest decoration behavior of black kites increases the potential nest visibility to aerial trespassers.

Although complex nest structures are common in nature, there are relatively few examples of the use of external objects to decorate breeding sites [7,15]. In most cases, ornamentation seems to act as an extended phenotype in mating contexts [7,15], as reported in some passerine species that place ornamental feathers on the nest exterior (e.g. [8,16,17]). In other cases, the use of (non-natural) elements has been suggested to have an antiparasitic function (e.g. cigarette butts in the nests of house sparrows, *Passer domesticus*, and house finches, *Carpodacus mexicanus*; [18]), or to provide thermal benefits (e.g. use of dung by white storks, *Ciconia ciconia*; [19]). However, evidence of birds using ornamental items as social signals is very scarce [10,11]. In Doñana, a large fraction of the black kite population decorates the nest with white materials (preferentially plastic, as shown experimentally by [10]), although the extent of decoration varies with age as only individuals in prime condition decorate their nests profusely. Furthermore, rates of conspecifics’ trespassing and egg predation increased when decoration was experimentally augmented, suggesting that dishonest signalers may incur great fitness costs [10]. As we now demonstrate here, using UAS to simulate a bird’s perspective when approaching a nest, the detectability of black kite nests increases with white decorations. Overall, these findings support the key assumption that such decorations increase nest conspicuousness from above and may thus serve as a reliable signal of threat against intruders, providing them with potential information on the social dominance, territory quality and fighting capabilities of the owners [10]. More crucially, the current results show that this information is available from far away, at distances from the nest that allow trespassers to gather knowledge before incurring the costs of a physical attack from the territory owners. Thus, signal design in this system seems consistent with the idea of a communication channel making a nest more visible from as far away as possible to an aerial intruder.

Interestingly, neither nest dimensions (length and width) nor its exposure from above (opening angles) influenced the probability and time to detection by our human observers (used as proxies). Since photographs were taken at different angles and heights, a possibility is that the fraction of the nest visible in each image may have not captured adequately all the tri-dimensional characteristics of the nests. Alternatively, the limited availability of nest sites may have affected the detectability of nests. In this saturated high-density population, many pairs have only 1–2 trees as available substrates [12] and, once a tree is chosen, few positions within the tree may be available for adequate nest-location given the constraints of local micro-climate or physical feasibility of construction (e.g. [20]).

Whatever the interaction with tree configuration, our results remark the use of white ornamentation as an efficient channel for transmission of information in space (i.e. towards the sky), since its effect may at least partially override the constraints imposed by available tree architecture (e.g. nests well concealed within the canopy had higher detection rates and lower latencies when decorated than when non-decorated). In fact, the effect of decoration was clearly noticeable to human eyes even in the pictures taken at the furthest distances analyzed and probably from much farther to actually prospecting kites, given their more acute vision [13]. Furthermore, the addition of plastic is probably less energetically demanding and time consuming than construction of larger, more conspicuous nests. It is also less risky than placing (large) nests in the external, more visible, but also weaker and more unstable upper branches of trees. In this regard, summer temperatures in Doñana may reach beyond 50°C in areas exposed to direct sunlight, which is clearly above the thermo-neutral zone for eggs and nestlings [21]. Thus, by decorating with white materials, kites might place their nests in sites with lower exposition (i.e. well within canopies) while still improving visibility. Also, the fact that nest decoration is placed at the peak of territory-intrusions just before laying, and declines as the breeding season progresses and temperatures increase [10], seems to rule out thermoregulation as the main function of this behavior.

### Potential limitations of the study

Although the use of humans observers is a popular approach in evolutionary studies to measure crypticity or conspicuousness (e.g. [22–24]), we recognize that the use of humans to rate pictures is an oversimplification of field conditions. However, it offers significant advantages by permitting image manipulation and controlled conditions, in terms of time, distance, and perspective, to assess detection of both decorated and undecorated nests. Furthermore, some experiments on crypsis have reported similar detection rates between avian and human predators (e.g. [22,24–26]). Humans and birds, including black kites, share a visually oriented sensory system and an excellent color and detail discrimination. If anything, diurnal raptors possess a higher visual acumen [27,28]. A main concern using human observers as black kite surrogates is that humans, unlike birds, have no ultra-violet (UV) sensitivity [29,30]. In the context of the present study, the use of humans to assess nest visibility may have led to underestimate the detection capacity of actual kites due to the UV component of the white decorations. Thus, given such potential differences in visual acuity and UV-perception, our results are likely to represent a conservative estimate of the detection advantage offered by nest ornamentation.

A second, potential limitation of the study was represented by the fact that the experimental material used to decorate the nests in our experiments was a standardized, new plastic bag. In our experience, kites tend to place new, shiny materials in the nest when first decorating it. For example, many kites readily collected new pieces of white plastic when these were experimentally offered in a previous study [10]. However, these materials will unavoidably become dirtier and less bright with time through physical wear, exposure to elements and contact with the nest contents. Thus, even if kites regularly refill their nests (F. Sergio, pers. obs.), our manipulation may have included brighter materials than experienced by an average intruder. In this sense, our measures may reflect the “ideal” decoration-decision by a kite at the moment of placing the item, later altered by environmental constraints.

Finally, we imposed high levels of nest decoration during the flights, so as to ensure an over-focus on individuals that intend to signal and most benefit from it. Due to financial and logistical constraints, we were unable to examine the level of visibility-enhancement offered by intermediate to low levels of decoration, associated with individuals that may strike a balance between an attempt to threaten conspecifics and to pass sufficiently unnoticed to avoid excessive intruder pressure.

In contrast with the above limitations, note that, given the proverbially extreme latero-vertical maneuverability of kite flight, a real kite intruder would be much more adept at finding the distance, flight altitude and angle that maximizes the visibility of an observed nest than our simplified, pre-planned, straight trajectories. This likely made our assessment unavoidably highly conservative and could have amply counter-balanced our over-decoration of kite nests. Thus, if on one hand we could have over-decorated our nests, on the other hand, we used a simulated intruder with very limited capacity of opportunistic aerial relocation for nest visibility-maximization from a distance. Because of the above uncontrollable factors, difficult to incorporate in an assessment of this kind, this study should be best seen as a clear, unavoidable simplification of an ideal situation, focused on physical signal transmission and only considered in conjunction and through its congruence with previous analyses of the same communication system [10].

## Conclusions

To our knowledge, this is the first study using UAS as a tool to simulate animals’ perception. Here we exploited the capability of UAS to act as an "eye in the sky" [31], but advances in the development of more types of miniaturized sensors may allow these systems to acquire further roles in ecology than simple aerial photography. Thus, it is now technologically possible to equip UAS with, among others, microphones, traps for microparticles and sensors of micro-meteorological variables, which may allow even more refined assessments of animals’ perceived and actual environments in the future [32].

The experimental simulation of nest inspection by a prospecting kite supported the idea of nest decoration by this species as a signal designed to enhance nest visibility and transmit information to flying territory trespassers. As a consequence, the latter could assess nest contents from a distance and within a shorter time span, thus minimizing the probability of an aggressive, physical reaction by the territory owners. Avoidance of physical fights for both the signaler and receiver may benefit both parties and contribute to the evolutionary maintenance of this communication system.

## Supporting Information

S1 File **Fig A.** Example image of the hexacopter during the experimental flights around black kite nests. **Table A.** Nest detection probability (A) and latency to nest detection (B) by human observers in relation to decoration treatment (decorated and non-decorated) in the approaching snapshots. Aside of the results shown in Table 1 of the main text, the models below included only the first ‘correct detection’ of each nest (i.e. 2 images per nest, one per treatment) instead of the 3 consecutive images per treatment. For example, if the subject A detected nest X in the 75 m distance photo, then all subsequent detections of nest X by A (regardless the decoration treatment) were not included in the analyses. See method for further details.
(DOCX)

## References

[1] Hansell M. Bird Nests and Construction Behaviour. Cambridge University Press, Cambridge, U.K.; 2005.

[2] Goodfellow P. Avian Architecture: How Birds Design, Engineer, and Build. Princeton University Press, Princeton, NJ.; 2011.

[3] Negro J, Blasco R, Rosell J, Finlayson C. Potential exploitation of avian resources by early humans: an overview from ethnographic and historical data. Quat Int. 2015; 10.1016/j.quaint.2015.09.034

[4] Eggers S, Griesser M, Nystrand M, Ekman J. Predation risk induces changes in nest-site selection and clutch size in the Siberian jay. Proc R Soc B Biol Sci. 2006;273: 701–706. 10.1098/rspb.2005.3373 PMC1560074 16608689

[5] Negro JJ, Hiraldo F. Nest-site selection and breeding success in the Lesser Kestrel Falco naumanni. Bird Study. 1993;40: 115–119. 10.1080/00063659309477136

[6] Petit KE, Petit L, Petit DR. Fecal sac removal: Do the pattern and distance of dispersal affect the chance of nest predation? Condor. 1989;91: 479–482.

[7] Mainwaring MC, Hartley IR, Lambrechts MM, Deeming DC. The design and function of birds’ nests. Ecol Evol. 2014;4: 3909–28. 10.1002/ece3.1054 25505520 PMC4242575

[8] Veiga JP, Polo V. Feathers at nests are potential female signals in the spotless starling. Biol Lett. 2005;1: 334–7. 10.1098/rsbl.2005.0329 17148200 PMC1617166

[9] Searcy W, Nowicki S. The evolution of animal communication: Reliability and deception in signaling systems. Princeton University Press; 2005.

[10] Sergio F, Blas J, Blanco G, Tanferna A, Lopez L, Lemus J a, et al Raptor Nest Decorations Are a Reliable Threat Against Conspecifics. Science. 2011;331: 327–330. 10.1126/science.1199422 21252345

[11] García-Navas V, Valera F, Griggio M. Nest decorations: an “extended” female badge of status? Anim Behav. Elsevier Ltd; 2015;99: 95–107. 10.1016/j.anbehav.2014.10.024

[12] Sergio F, Blas J, López L, Tanferna A, Díaz-Delgado R, Donázar J a., et al Coping with uncertainty: breeding adjustments to an unpredictable environment in an opportunistic raptor. Oecologia. 2011;166: 79–90. 10.1007/s00442-010-1795-x 20953963

[13] Shlaer R. An Eagle’s Eye: Quality of the Retinal Image. Science (80-). 1972;176: 920–922. 10.1126/science.176.4037.920 5033635

[14] Kuznetsova A, Brockhoff PB, Christensen RHB. Package lmerTest [Internet]. 2015. Available: https://cran.r-project.org/web/packages/lmerTest/index.html

[15] Schaedelin FC, Taborsky M. Extended phenotypes as signals. Biol Rev Camb Philos Soc. 2009;84: 293–313. 10.1111/j.1469-185X.2008.00075.x 19382933

[16] Sanz JJ, Garcia-Navas V. Nest ornamentation in blue tits: is feather carrying ability a male status signal? Behav Ecol. 2011;22: 240–247. 10.1093/beheco/arq199

[17] Brouwer L, Komdeur J. Green nesting material has a function in mate attraction in the European starling. Anim Behav. 2004;67: 539–548. 10.1016/j.anbehav.2003.07.005

[18] Suárez-Rodríguez M, López-Rull I, Garcia CM. Incorporation of cigarette butts into nests reduces nest ectoparasite load in urban birds: new ingredients for an old recipe? Biol Lett. 2013;9: 20120931 10.1098/rsbl.2012.0931 23221874 PMC3565511

[19] Tortosa FS, Villafuerte R. Effect of nest microclimate on effective endothermy in White Stork Ciconia ciconia nestlings. Bird Study. 1999;46: 336–341. 10.1080/00063659909461147

[20] Viñuela J, Sunyer C. Nest orientation and hatching success of black kites in Spain. Ibis (Lond 1859). 1992;134: 340–345.

[21] Viñuela J. Opposing selective pressures on hatching asynchrony: egg viability, brood reduction, and nestling growth. Behav Ecol Sociobiol. 2000;48: 333–343. 10.1007/s002650000245

[22] Beatty CD, Bain RS, Sherratt TN. The evolution of aggregation in profitable and unprofitable prey. Anim Behav. 2005;70: 199–208. 10.1016/j.anbehav.2004.09.023

[23] Stevens M. Predator perception and the interrelation between different forms of protective coloration. Proc R Soc B Biol Sci. 2007;274: 1457–64. 10.1098/rspb.2007.0220 PMC1950298 17426012

[24] Penney HD, Hassall C, Skevington JH, Abbott KR, Sherratt TN. A comparative analysis of the evolution of imperfect mimicry. Nature. Nature Publishing Group; 2012;483: 461–464. 10.1038/nature10961 22437614

[25] Dittrich W, Gilbert F, Green P, Mcgregor P, Grewcock D. Imperfect mimicry : a pigeon ‘ s perspective. Proc R Soc B Biol Sci. 1993;251: 195–200.

[26] Karpestam E, Merilaita S, Forsman A. Detection experiments with humans implicate visual predation as a driver of colour polymorphism dynamics in pygmy grasshoppers. BMC Ecol. 2013;13: 17 10.1186/1472-6785-13-17 23639215 PMC3648452

[27] Güntürkün O. Structure and functions of the eye. Sturkie’s Avian Physiology. 5th Editio Academic Press, San Diego; 1998.

[28] Brooke MDL, Hanley S, Laughlin SB. The scaling of eye size with body mass in birds. Proc R Soc B Biol Sci. 1999;266: 405 10.1098/rspb.1999.0652

[29] Cuthill I, Partridge J, Bennett A, Church S, Hart N, S H. Ultraviolet vision in birds. Adv Study Behav. 2000;29: 159–214.

[30] Bennett A, Cuthill I. Ultraviolet vision in birds: What is its function? Vision Res. 1994;34: 1471–1478. 10.1016/0042-6989(94)90149-X 8023459

[31] Rodríguez A, Negro JJ, Mulero M, Rodríguez C, Hernández-Pliego J, Bustamante J. The Eye in the Sky: Combined Use of Unmanned Aerial Systems and GPS Data Loggers for Ecological Research and Conservation of Small Birds. Renton K, editor. PLoS One. 2012;7: e50336 10.1371/journal.pone.0050336 23239979 PMC3519840

[32] Anderson K, Gaston KJ. Lightweight unmanned aerial vehicles will revolutionize spatial ecology. Front Ecol Environ. 2013;11: 138–146. 10.1890/120150

