# Supplementary material for: Decoration Increases the Conspicuousness of Raptor Nests
Source: PLoS One. 2016 Jul 25;11(7):e0157440. doi: 10.1371/journal.pone.0157440 (PMC4959696; doi:10.1371/journal.pone.0157440)
Supplement: S1 File — Fig A. Example image of the hexacopter during the experimental flights around black kite nests. Table A. Nest detection probability (A) and latency to nest detection (B) by human observers in relation to decoration treatment (decorated and non-decorated) in the approaching snapshots. Aside of the results shown in Table 1 of the main text, the models below included only the first ‘correct detection’ of each nest (i.e. 2 images per nest, one per treatment) instead of the 3 consecutive images per treatment. For example, if the subject A detected nest X in the 75 m distance photo, then all subsequent detections of nest X by A (regardless the decoration treatment) were not included in the analyses. See method for further details. (DOCX) [file pone.0157440.s001.docx]

Supplementary Material

**S1 Fig A.**


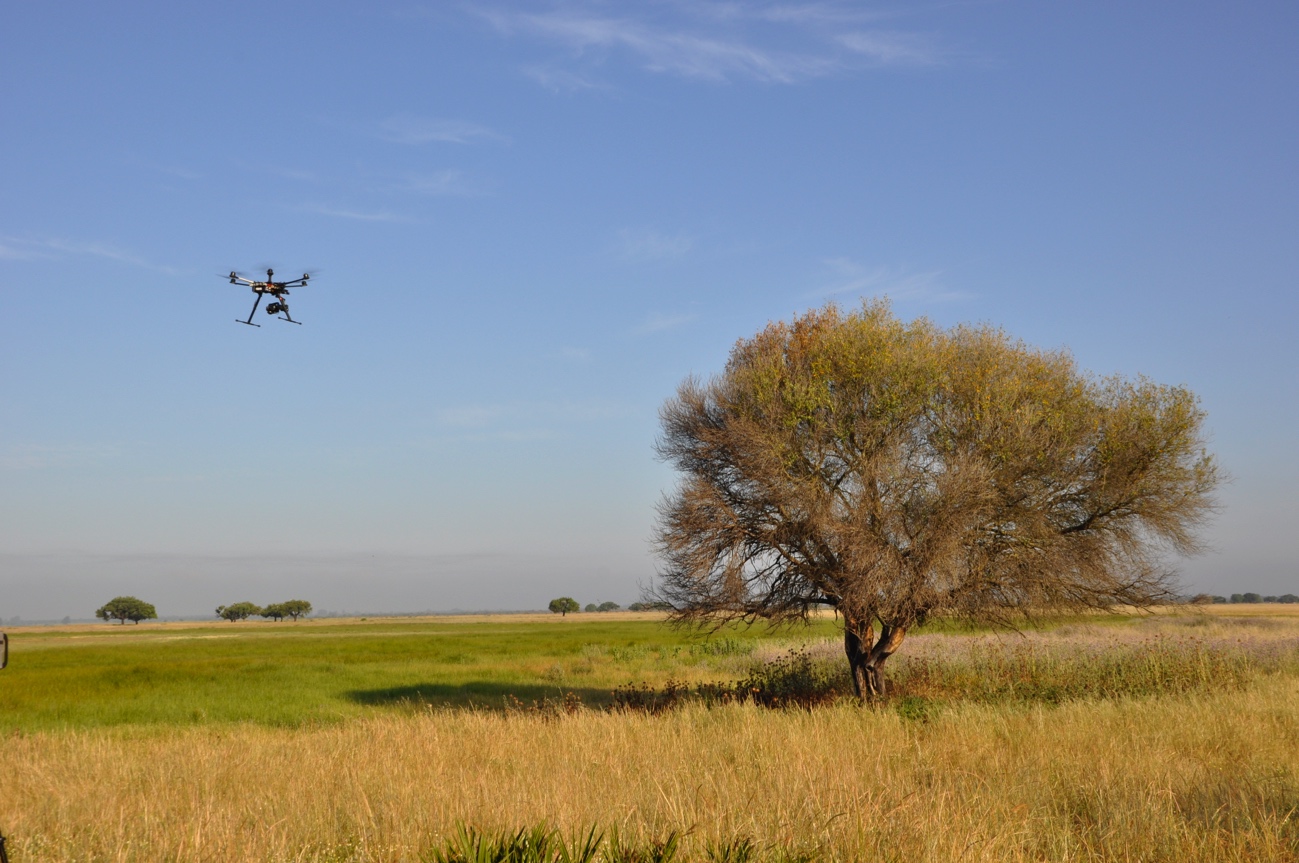


**Nest**

**S1 Table A.**

| A | **Estimate** | **Std. Error** | **z value** | **P** |
| --- | --- | --- | --- | --- |
| Intercept | -1.189 | 0.228 | -5.19 | <0.001 |
| Treatment (decorated) | 3.437 | 0.298 | 11.53 | <0.001 |
| B | **Estimate** | **Std. Error** | **t value** | **P** |
| Intercept | 1.554 | 0.047 | 32.71 | <0.001 |
| Treatment (decorated) | -0.295 | 0.04 | -7.29 | <0.001 |

|  |  |  |  |
| --- | --- | --- | --- |
